# Training and Experiential Factors Associated With Transcranial Magnetic Stimulation Referral Readiness Among Psychiatric Providers

**DOI:** 10.1002/brb3.71876

**Published:** 2026-10-30

**Authors:** Aneesh Rahangdale, Lauren Ferraro, Gracyn Wood, Christopher Wadsworth, Eileen Venable

**Affiliations:** ^1^ College of Medicine/HCA Florida Capital Hospital Psychiatry University of Central Florida Tallahassee Florida USA; ^2^ Maclay School Tallahassee Florida USA; ^3^ Florida State University Tallahassee Florida USA; ^4^ University of California San Francisco San Francisco California USA; ^5^ Boston Children's Hospital Harvard University, Boston MA USA

**Keywords:** psychiatric providers, referral readiness, treatment‐resistant depression, transcranial magnetic stimulation

## Abstract

**Objective:**

Transcranial magnetic stimulation (TMS) is an established treatment for patients with treatment‐resistant depression (TRD); however, barriers remain in clinical practice. This study examined how psychiatric clinicians view TMS and evaluated whether training exposure and prior experience were associated with their readiness to refer patients for treatment.

**Methods:**

We conducted a cross‐sectional survey of psychiatric providers in Florida, including attending psychiatrists, psychiatry residents, and psychiatric nurse practitioners. The survey assessed beliefs about TMS effectiveness, comfort discussing and referring the treatment, familiarity with referral processes, and perceived barriers. Responses were dichotomized to reflect endorsement, and group differences were analyzed using chi‐square or Fisher exact tests.

**Results:**

Ninety‐four clinicians completed the survey. Conceptual endorsement of TMS was high, with more than 90% agreeing that it is an effective treatment for depression. Operational readiness was more variable, particularly for referral comfort, counseling confidence, and knowledge of referral pathways. Prior TMS training and prior TMS recommendation experience were associated with greater comfort referring patients, greater confidence counseling patients, and higher awareness of referral steps. Psychiatry residents reported lower referral‐pathway awareness than attending psychiatrists. Insurance coverage and treatment costs were frequently reported barriers, and lack of training was a commonly endorsed modifiable barrier.

**Conclusions:**

While psychiatric providers widely accepted TMS conceptually, operational readiness to counsel and refer was more variable. Training exposure and prior TMS recommendation experience were associated with greater confidence, comfort, and familiarity with referral pathways, although causality cannot be inferred from this cross‐sectional design. These findings suggest that efforts to improve utilization should emphasize building operational competence and confidence, along with clearer referral workflows and accessible system‐level processes.

## Graphical abstract

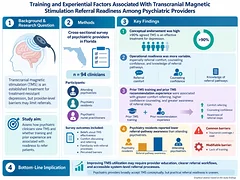

## 1 Introduction

### 1.1 Background and Significance

Major depressive disorder (MDD) is a leading cause of disability worldwide and affects a substantial proportion of adults over the course of life (Liu et al. 2020; McIntyre et al. 2023). Despite advances in medication and psychotherapy, many patients do not achieve adequate relief with first‐line treatments. The STAR*D trial showed that only about one‐third of patients remit after an initial antidepressant trial, with progressively lower remission rates across subsequent steps (Rush et al. 2006). More recent work suggests cumulative remission may be even lower than first reported (Pigott et al. 2023). Contemporary reviews indicate that treatment‐resistant depression (TRD) affects a substantial minority of patients with MDD, often estimated at roughly 20%–30% depending on the definition and population studied (Fava 2003; McIntyre et al. 2023). For definitional purposes, TRD is typically described as nonresponse to at least two adequate antidepressant trials from different classes (Fava 2003; McIntyre et al. 2023).

The burden of TRD is substantial. Patients experience greater functional impairment, elevated suicide risk, and increased healthcare utilization, including emergency department visits and hospitalizations (Zhdanava et al. 2020; Mrazek et al. 2014). Annual healthcare costs are markedly higher, with direct expenditures estimated to exceed $17,000 per patient and total costs surpassing $20,000 per year (Zhdanava et al. 2020). The emotional and financial cost of TRD on patients and society highlights the need for effective, scalable, and accessible treatments for individuals who do not respond to first‐line pharmacotherapy and psychotherapy.

### 1.2 Transcranial Magnetic Stimulation as an Evidence‐Based Treatment

Transcranial magnetic stimulation (TMS) has emerged as a well‐supported treatment option for TRD (Gaynes et al. 2014; Vida et al. 2023; Chen et al. 2025; Trapp et al. 2025). TMS is a noninvasive neuromodulation technique that uses focused magnetic fields to modulate cortical activity, most commonly targeting the left dorsolateral prefrontal cortex implicated in mood regulation (Gaynes et al. 2014). The U.S. Food and Drug Administration first cleared the NeuroStar TMS System in 2008 for adult patients with MDD who failed to achieve satisfactory improvement from one prior antidepressant trial in the current episode (US Food and Drug Administration 2008). Multiple randomized trials and meta‐analyses demonstrate that repetitive TMS (rTMS) produces clinically meaningful improvements compared with sham (Gaynes et al. 2014; Vida et al. 2023; Avery et al. 2006). The safety profile is favorable, with adverse effects generally limited to transient headache and scalp discomfort. Unlike electroconvulsive therapy, TMS does not intentionally induce seizures or require anesthesia, although rare seizures are a recognized adverse event (O'Reardon et al. 2007). Yet, despite more than 15 years of clinical use, TMS remains underutilized in routine practice (Stern et al. 2016).

### 1.3 The Role of Healthcare Provider Perceptions in Utilization

Clinicians serve as the primary gatekeepers to neuromodulation services, and their knowledge, attitudes, and familiarity with referral processes may be associated with whether eligible patients access TMS (Stern et al. 2016; Mehalick and Laje 2020). Today's depression care involves attending psychiatrists, psychiatry residents, and psychiatric nurse practitioners, whose training, scope, and exposure to TMS can differ meaningfully. These differences likely contribute to variation in familiarity with referral logistics and patient counseling across settings.

### 1.4 Existing Evidence on Knowledge Gaps and Barriers

Surveys in the United States and internationally generally show favorable views of TMS efficacy and safety among psychiatrists but highlight persistent gaps in knowledge and limited training exposure (Stern et al. 2016; Deng et al. 2020; AlHadi et al. 2017; Yonel and Abdelghani 2020; Mehalick and Laje 2020; Albishi et al. 2024). Financial and insurance‐related issues further complicate the adoption of TMS. Provider uncertainty around insurance coverage, prior authorization, and administrative burden can deter referrals even when clinically indicated (Cortright et al. 2024; Taylor et al. 2025). System‐level factors, including geographic disparities and limited availability, particularly in rural or underserved regions, also constrain access (Basiru et al. 2024; Goldbloom and Gratzer 2020).

### 1.5 Rationale and Study Objectives

Despite the growing recognition of TMS as an effective treatment for TRD, important gaps remain in understanding how provider perceptions vary across disciplines, training levels, and experience. Existing studies have largely focused on psychiatrists, limiting the ability to develop comprehensive, provider‐specific implementation strategies.

The present study addresses these gaps by systematically examining healthcare provider perceptions of TMS across multiple provider types involved in adult depression care. Specifically, this study aims to:

1. assess provider attitudes toward TMS effectiveness
2. evaluate confidence in counseling patients and comfort with referral
3. examine awareness of referral pathways
4. identify perceived barriers to TMS utilization

This study further examines how these outcomes vary by provider role, training exposure, and prior TMS recommendation experience.

## 2 Materials and Methods

### 2.1 Study Design and Conceptual Framework

We conducted a cross‐sectional, descriptive survey to examine how psychiatric providers perceive TMS as a treatment for TRD. The survey assessed several domains: attitudes toward TMS effectiveness, confidence in counseling patients, comfort referring patients for TMS, familiarity with referral pathways, and perceived barriers to use. We also explored how these perceptions differed based on provider characteristics, prior training, and prior recommendation of TMS.

Survey development drew upon the Knowledge, Attitudes, and Practice (KAP) model, which frames clinical behavior as shaped by what clinicians know, believe, and feel capable of doing. Elements of the Consolidated Framework for Implementation Research (CFIR) were used to guide item development, particularly related to provider attributes and factors such as training opportunities, institutional support, and referral logistics. CFIR elements were applied conceptually for survey structure and were not used to generate formal implementation scores.

### 2.2 Setting and Participants

The study was conducted in Florida and targeted healthcare providers involved in the care of adults with depressive disorders. Three groups were included:

1. psychiatry faculty affiliated with HCA (Hospital Corporation of America) Healthcare facilities
2. psychiatry residents in HCA‐affiliated residency programs
3. psychiatric nurse practitioners working in outpatient mental health settings in Tallahassee

Faculty and residents were recruited through institutional email lists. Psychiatric nurse practitioners were recruited through peer dissemination among outpatient psychiatric clinicians in Tallahassee.

These groups were selected because they commonly evaluate and manage adults with MDD and may identify candidates for TMS referral in routine clinical practice. Residents were included because referral habits and neuromodulation exposure often develop during training.

### 2.3 Eligibility Criteria

Participants were eligible if they:

1. were actively practicing in Florida

Florida practice was required because recruitment occurred through Florida‐based institutional and professional networks, and the study aimed to characterize referral readiness within this regional practice context.

1. Had managed adult depression within the prior 12 months
2. Could complete an English‐language survey
3. Provided electronic informed consent

Participants were excluded if they did not have current direct patient‐care responsibilities or practiced exclusively in pediatric settings.

### 2.4 Sampling Strategy and Recruitment

A convenience sampling approach was used. Faculty and residents were recruited through institutional email lists. Psychiatric nurse practitioners were recruited through peer dissemination among outpatient psychiatric clinicians in Tallahassee. The recruitment messages described the study purpose, estimated completion time, voluntary participation, and included a secure survey link. No incentives were provided to complete the survey.

### 2.5 Survey Instrument Development

The survey was developed through a multi‐step process. A targeted literature review informed the initial item pool, highlighting existing surveys and commonly reported barriers related to TMS and other neuromodulation interventions. Items assessed include:

• provider demographics and practice characteristics
• prior exposure to TMS (training and recommendation experience)
• attitudes toward TMS effectiveness
• confidence counseling patients about TMS
• comfort with TMS referral
• awareness of referral processes
• barriers to TMS implementation
• educational needs related to neuromodulation

Question formats included categorical items, multiple‐choice questions, and five‐point Likert scales. The instrument was developed for descriptive and exploratory purposes and was reviewed for face and content validity by psychiatrists, residents, and psychiatric nurse practitioners. Formal psychometric validation was not performed.

### 2.6 Data Collection Procedures

Data were collected using Qualtrics, a secure, web‐based survey platform. Participants provided electronic informed consent before accessing the survey. Surveys were anonymous, and no identifiable information was collected. The survey remained open for 8 weeks, with periodic reminders sent to encourage responses. Participants could complete the survey on desktop or mobile devices.

### 2.7 Data Management and Quality Assurance

All data were stored on secure servers with restricted access. Access to the survey dataset was restricted to study team members responsible for data management and analysis. Surveys with substantial missing data on primary outcome measures were excluded from inferential analysis. Data were fully de‐identified in accordance with HIPAA Safe Harbor standards.

### 2.8 Outcome Measures

Primary outcomes included:

• attitudes toward TMS effectiveness
• confidence in counseling patients regarding TMS
• comfort referring patients for TMS
• awareness of TMS referral pathways

Secondary outcomes included perceived barriers related to:

• insurance coverage and cost
• training and educational exposure
• access and availability of TMS services

For inferential analyses, agreement‐based items were dichotomized by combining “somewhat agree” and “strongly agree” versus all other responses. The referral comfort item used a comfort scale and was dichotomized as “somewhat comfortable” or “extremely comfortable” versus all other responses. Endorsement of prior TMS education/training was defined as selecting “somewhat agree” or “strongly agree” on that item, with all other responses categorized as non‐endorsement. Given the modest sample size and the goal of identifying clinically interpretable endorsement patterns, dichotomization was used for the primary analyses, whereas full five‐point Likert distributions were retained in the figures and tables in the Supporting Information. We recognize that dichotomization reduces granularity and may reduce statistical power.

### 2.9 Statistical Analysis

Analyses followed a pre‐specified plan emphasizing descriptive statistics and group comparisons. Categorical variables were summarized as frequencies and percentages. Associations between provider characteristics (provider type, prior training, and prior TMS recommendation experience) and survey outcomes were examined using chi‐square tests when assumptions were met or Fisher exact tests when cell sizes were small. Odds ratios (ORs) with 95% confidence intervals (CIs) were calculated to quantify both the strength and precision of associations. Agreement‐based items were dichotomized as endorsement (“somewhat agree” or “strongly agree”) versus all other responses. The referral comfort item used a comfort scale and was dichotomized as “somewhat comfortable” or “extremely comfortable” versus all other responses. Given the modest sample size and the goal of identifying clinically interpretable endorsement patterns, dichotomization was used for the primary analyses, while full five‐point response distributions were retained in the figures and tables in the Supporting Information. Statistical significance was defined as *p* <0.05. Because this was an exploratory, descriptive survey designed to identify patterns of association rather than test a single confirmatory hypothesis, we did not apply a formal correction for multiple comparisons. Findings, particularly *p*‐values near 0.05, should therefore be interpreted as hypothesis‐generating. Adjusted multivariable models were not performed because the study was designed as a descriptive analysis, subgroup counts were modest, and the number of non‐endorsed outcomes was limited in several strata, raising concern that adjusted estimates would be unstable and potentially misleading. Interpretation focused on the direction and magnitude of associations rather than causal inference. Findings, particularly *p*‐values near 0.05, should therefore be interpreted as hypothesis‐generating.

### 2.10 Ethics Approval Statement

This study was reviewed through HCA Healthcare's centralized research compliance process and was determined to meet criteria for exemption from Institutional Review Board oversight under 45 CFR 46.104(d)(2) (reference number 2025‐1388). Exemption was granted due to the anonymous nature of the survey and use of de‐identified data. Participation was voluntary, and the study posed minimal risk. All procedures were conducted in accordance with applicable ethical standards and the Declaration of Helsinki.

## 3 Results

### 3.1 Sample Characteristics

A total of 375 surveys were distributed or disseminated across three provider groups: attending psychiatrists (*n* = 183), psychiatry residents (*n* = 167), and psychiatric nurse practitioners (*n* = 25). Surveys were distributed to attending psychiatrists and psychiatry residents and disseminated to psychiatric nurse practitioners through peer dissemination. Ninety‐nine surveys were completed (99/375 = 26.4%), and 94 contained fully analyzable responses (94/375 = 25.1%). These respondents represented attending psychiatrists (*n* = 31), psychiatric nurse practitioners (*n* = 17), and psychiatry residents (*n* = 46). Participant demographics, practice characteristics, and prior TMS exposure are summarized in Table 1. Subgroup comparisons were performed descriptively and inferentially where feasible, although small subgroup sizes limit precision. The higher number of resident respondents reflects the participation of multiple residency programs in the survey sample.

**TABLE 1:** Participant characteristics.

Characteristic	Overall sample (*N* = 94)
Provider role	Psychiatrist, 31 (33.0%); psychiatry resident, 46 (48.9%); psychiatric nurse practitioner, 17 (18.1%)
Years in practice	0–5 years, 58 (61.7%); 6–10 years, 9 (9.6%); 11–15 years, 4 (4.3%); 16–20 years, 5 (5.3%); >20 years, 18 (19.1%)
Practice setting	Hospital system, 51 (54.3%); private practice, 25 (26.6%); academic medical center, 12 (12.8%); community health center, 6 (6.4%)
Practice location	Suburban, 56 (59.6%); urban, 31 (33.0%); rural, 7 (7.4%)
Monthly depression patient volume	<10, 1 (1.1%); 10–25, 13 (13.8%); 26–50, 34 (36.2%); 51–100, 30 (31.9%); >100, 16 (17.0%)
Prior TMS training	Yes, 49 (52.1%); no, 45 (47.9%)
Prior TMS recommendation experience	Yes, 71 (75.5%); no, 23 (24.5%)

*Note*: Participant demographics, practice characteristics, and prior TMS exposure among the 94 analyzable respondents are shown. Values are presented as n (%) unless otherwise noted.

### 3.2 Overall Provider Perceptions of TMS

Across the full cohort, respondents expressed strongly positive views of TMS. Overall, 91.5% agreed that TMS is an effective treatment for depression, and most clinicians reported feeling reasonably confident discussing the treatment with patients (79.8%) and referring eligible individuals for TMS (80.9%). Together, these findings suggest broad acceptance of TMS and general comfort with its role in clinical practice.

In contrast, familiarity with the practical steps involved in making a referral was notably lower. Just over half of respondents (53.2%) indicated that they knew how to initiate a TMS referral, highlighting a gap between conceptual endorsement and operational readiness.

Full five‐point response distributions for the main survey items are provided in Table S1. As expected, ratings of treatment effectiveness clustered heavily toward agreement, with relatively few neutral or negative responses. However, responses related to counseling confidence, referral comfort, and especially referral‐pathway awareness showed greater variability, with many clinicians selecting neutral or uncertain options. This pattern suggests that uncertainty about logistical aspects of TMS referral remains common even among providers who view the treatment favorably.

### 3.3 Comparisons by Provider Type

Comparisons across provider types are summarized in Table 2 and shown in Figure 1. Positive attitudes toward the effectiveness of TMS were high in all groups, with no significant differences between psychiatrists, psychiatric nurse practitioners, and psychiatry residents (*χ* ^2^ = 0.21, df = 2, *p* = 0.902, Cramér's *V* = 0.05). Referral comfort and confidence counseling patients also did not differ significantly by provider type.

**TABLE 2:** Provider type comparison.

Provider group	Positive attitude (agree) (%)	Referral comfort (%)	Counseling confidence (agree) (%)	Referral‐pathway awareness (agree) (%)	*n*
Psychiatrist	90.3	87.1	87.1	67.7	31
Psychiatric nurse practitioner	94.1	88.2	76.5	64.7	17
Psychiatry resident	91.3	73.9	76.1	39.1	46

*Note*: Percentage of psychiatrists, psychiatric nurse practitioners, and psychiatry residents endorsing positive attitudes toward TMS effectiveness, referral comfort, counseling confidence, and referral‐pathway awareness. Overall group differences were assessed using chi‐square tests (attitude: *χ* ^2^ = 0.21, p = .902; referral comfort: *χ* ^2^ = 2.81, *p* = 0.245; counseling confidence: *χ* ^2^ = 1.53, *p* = 0.465; referral‐pathway awareness: *χ* ^2^ = 7.19, *p* = 0.027).

**FIGURE 1:**
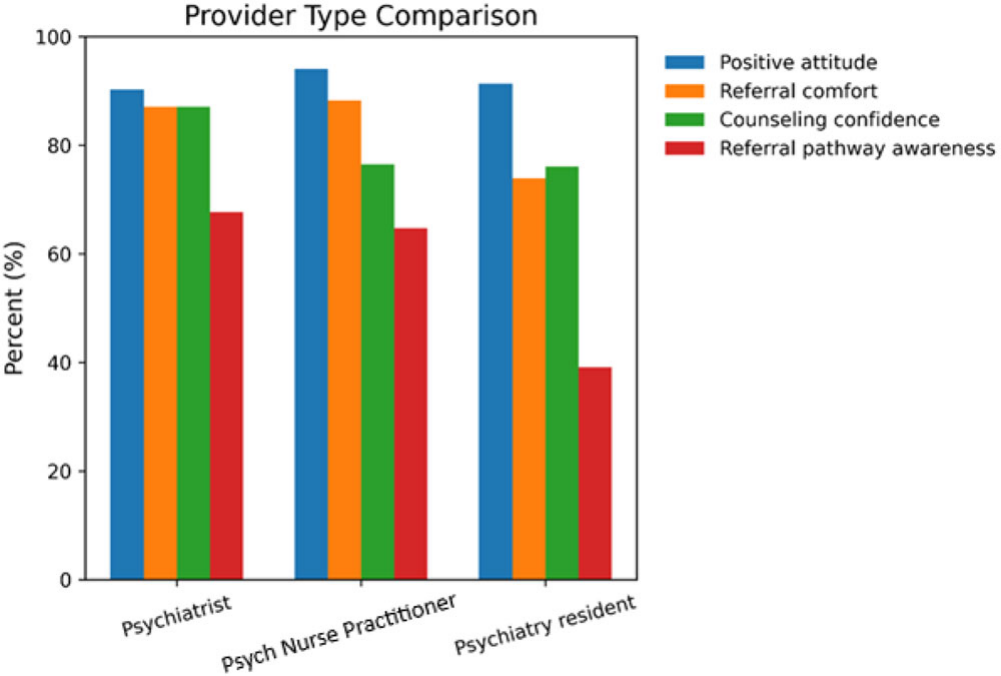
Provider type comparison. Percentage of respondents endorsing positive attitude toward TMS effectiveness, referral comfort, counseling confidence, and referral‐pathway awareness across provider groups (attending psychiatrists, psychiatric nurse practitioner, and psychiatry residents). Attitudes were uniformly high, whereas referral‐pathway awareness was notably lower among residents.

Referral‐pathway awareness differed by provider type in unadjusted analysis, but this exploratory finding should be interpreted cautiously given multiple comparisons and subgroup size. Psychiatry residents reported lower awareness (39.1%) compared with attending psychiatrists (67.7%) and psychiatric nurse practitioners (64.7%).

Figure 1 highlights these patterns, showing consistent attitudes and confidence across provider groups alongside a marked gap in referral‐pathway awareness among residents.

### 3.4 Comparisons by TMS Training Status

Outcomes stratified by prior TMS education or training are summarized in Table 3 and Figure 2. Providers who had prior TMS education or training demonstrated higher levels of implementation readiness across all evaluated domains. Referral comfort differed significantly by training status: 93.9% of trained providers reported feeling comfortable referring patients for TMS, compared with 66.7% of those without training (*χ* ^2^ = 11.22, *p* < 0.001; OR = 7.67, 95% CI 2.04–28.76; *φ* = 0.35).

**TABLE 3:** Outcomes by TMS training status.

Outcome	Yes % (*n* = 49)	No % (*n* = 45)	Test	Statistic	OR, 95% CI	Effect (phi/V)	*p* value
Positive attitude (agree)	98.0 (48/49)	84.4 (38/45)	Fisher exact	—	8.84 (1.04–75.38)	—	0.026
Referral comfort (somewhat/extremely)	93.9 (46/49)	66.7 (30/45)	Chi‐square	11.22	7.67 (2.04–28.76)	0.35	<0.001
Counseling confidence (agree)	93.9 (46/49)	64.4 (29/45)	Chi‐square	12.60	8.46 (2.26–31.60)	0.37	<0.001
Referral pathway awareness (agree)	67.3 (33/49)	37.8 (17/45)	Chi‐square	8.24	3.40 (1.45–7.93)	0.30	0.004

*Note*: Comparison of outcomes between providers with and without prior TMS training, including positive attitudes toward TMS effectiveness, referral comfort, counseling confidence, and referral‐pathway awareness. Odds ratios (ORs) with 95% confidence intervals (CIs) and effect sizes (φ/V) are shown for each outcome. Fisher's exact test was used when expected cell counts were small; chi‐square tests were used otherwise. Providers with training demonstrated higher endorsement across outcomes, with large effects for referral comfort (OR = 7.67, 95% CI 2.04–28.76; *φ* = 0.35) and counseling confidence (OR = 8.46, 95% CI 2.26–31.60; *φ* = 0.37). CIs were wide for some estimates, reflecting small cell counts and high proportions.

**FIGURE 2:**
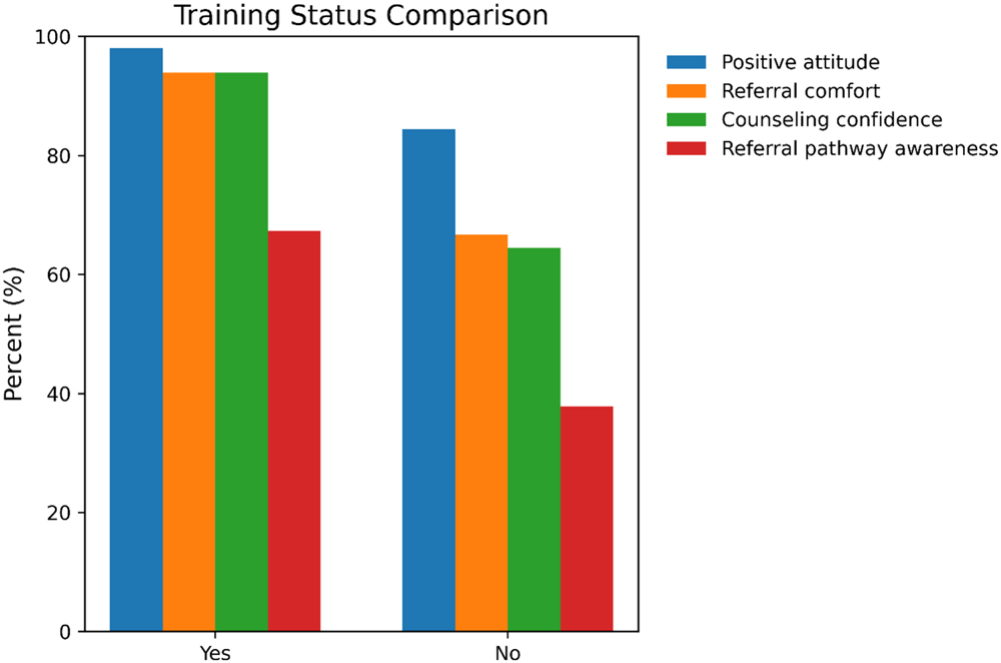
Outcomes by prior TMS training. Comparison of positive attitudes, referral comfort, counseling confidence, and referral‐pathway awareness between providers with and without prior TMS training. Providers endorsing prior TMS education/training reported higher readiness‐related outcomes across all domains, with the largest differences observed for referral comfort and counseling confidence.

A similar pattern emerged for counseling confidence. Nearly all trained providers (93.9%) felt confident counseling patients about TMS, whereas confidence was markedly lower among untrained providers (64.4%; *χ* ^2^ = 12.60, *p* < 0.001; OR = 8.46, 95% CI 2.26–31.60; *φ* = 0.37). Awareness of referral pathways also differed significantly, with 67.3% of the trained providers endorsing awareness compared with 37.8% of the untrained providers (*χ* ^2^ = 8.24, *p* = 0.004; OR = 3.40, 95% CI 1.45–7.93; *φ* = 0.30).

Attitudes toward TMS effectiveness were high in both groups but modestly higher among those with training (98.0% vs. 84.4%). This difference reached statistical significance on Fisher exact testing (*p* = 0.026), though the magnitude was smaller than the differences observed for referral comfort, counseling confidence, and referral‐pathway awareness.

### 3.5 Comparisons by Prior TMS Recommendation Experience

Comparisons based on prior TMS recommendation experience are summarized in Table 4. Prior TMS recommendation experience showed the largest observed associations with implementation‐related outcomes across all domains. Providers who had previously recommended TMS for a patient were far more likely to report comfort with the referral process than those without such experience (94.4% vs. 39.1%; Fisher exact test, *p* < 0.001; OR = 26.06, 95% CI 7.02–96.67), representing a large unadjusted association.

**TABLE 4:** Outcomes by prior TMS recommendation experience.

Outcome	Yes % (*n* = 71)	No % (*n* = 23)	Test	Statistic	OR, 95% CI	Effect (phi/V)	*p* value
Positive attitude (agree)	95.8 (68/71)	78.3 (18/23)	Fisher exact	—	6.30 (1.27–31.16)	—	0.020
Referral comfort (somewhat/extremely)	94.4 (67/71)	39.1 (9/23)	Fisher exact	—	26.06 (7.02–96.67)	—	<0.001
Counseling confidence (agree)	93.0 (66/71)	39.1 (9/23)	Fisher exact	—	20.53 (5.97–70.67)	—	<0.001
Referral pathway awareness (agree)	64.8 (46/71)	17.4 (4/23)	Chi‐square	15.67	8.74 (2.68–28.53)	0.41	<0.001

*Note*: Comparison of outcomes between providers with and without prior TMS recommendation experience, including positive attitudes toward TMS effectiveness, referral comfort, counseling confidence, and referral‐pathway awareness. Odds ratios (ORs) with 95% confidence intervals (CIs) are shown for each outcome. Fisher's exact test was used when expected cell counts were small; chi‐square tests were used otherwise. Providers with prior TMS recommendation experience showed markedly higher referral comfort (OR = 26.06, 95% CI 7.02–96.67) and counseling confidence (OR = 20.53, 95% CI 5.97–70.67), and greater referral‐pathway awareness with a large effect size (*χ* ^2^ = 15.67; OR = 8.74, 95% CI 2.68–28.53; *φ* = 0.41). CIs were wide for some estimates, reflecting small cell counts and high proportions.

Confidence in counseling patients followed a similar pattern. Nearly all providers with prior TMS recommendation experience (93.0%) reported feeling confident discussing TMS with patients, compared with 39.1% among those without such experience (Fisher exact test, *p* < 0.001; OR = 20.53, 95% CI 5.97–70.67). Referral‐pathway awareness also differed sharply between groups (64.8% vs. 17.4%; *χ* ^2^ = 15.67, *p* < 0.001; OR = 8.74, 95% CI 2.68–28.53; *φ* = 0.41), representing a large effect size.

Although attitudes toward TMS effectiveness were high in both groups, providers with prior TMS recommendation experience reported significantly higher endorsement (95.8% vs. 78.3%; *p* = 0.020). As with the training‐based comparisons, the size of this difference was smaller than the differences observed for referral comfort, counseling confidence, and referral‐pathway awareness.

### 3.6 Perceived Barriers to TMS Utilization

Perceived barriers by training status are summarized in Table 5. Insurance and cost related barriers were frequently endorsed across the sample and did not differ significantly by training background, suggesting these concerns were shared regardless of prior exposure to TMS.

**TABLE 5:** Perceived barriers by training status.

Training status	Insurance/Cost barrier (%)	Insurance/cost barrier (*p* value)	Training need barrier (%)	Training need barrier (*p* value)	Access/availability barrier (%)	Access/availability barrier (*p* value)	Institutional support barrier (%)	Institutional support barrier (*p* value)	Patient interest barrier (%)	Patient interest barrier (*p* value)	*n*
No	64.4	0.241	82.2	<0.001	35.6	0.468	15.6	0.082	42.2	0.212	45
Yes	75.5	0.241	36.7	<0.001	28.6	0.468	4.1	0.082	55.1	0.212	49

*Note*: Distribution of perceived barriers, including insurance/cost, training needs, access/availability, institutional support, and patient interest, among providers with and without prior TMS training. Group differences were assessed with chi‐square tests or Fisher exact tests as appropriate; *p*‐values are shown for each barrier domain. Lack of training was more frequently endorsed as a barrier among untrained providers (OR = 0.13, 95% CI 0.05–0.36, the comparison represents respondents with prior training relative to those without prior training), whereas insurance and cost concerns were common across both groups.

In contrast, training‐related barriers showed clear differences. Providers without prior TMS training were far more likely to report lack of training as a barrier than those with training (82.2% vs. 36.7%; *χ* ^2^ = 19.99, *p* < 0.001; OR = 0.13, 95% CI 0.05–0.36; *φ* = 0.46; the OR comparison represents respondents with prior training relative to those without prior training). Access‐ and availability‐related barriers were reported by a smaller proportion of respondents overall and did not differ meaningfully by training status. Institutional support barriers were uncommon in both groups but were somewhat more frequently endorsed by untrained providers. Perceived barriers related to patient interest showed no significant differences between groups.

## 4 Discussion

In this cross‐sectional survey, clinicians expressed broadly positive views of TMS, indicating high conceptual endorsement across the sample. In contrast, operational readiness was more variable, particularly for referral‐pathway awareness, counseling confidence, and referral comfort. Together, these findings suggest that endorsement of TMS as an effective treatment does not necessarily coincide with confidence in how to discuss, navigate, or initiate referral in routine care.

The ORs reported for the primary outcome comparisons and the training‐related barrier analysis were accompanied by wide CIs, reflecting the modest sample size and small cell counts in some comparisons. Despite this imprecision, the direction and magnitude of associations were consistent across outcomes, supporting a relationship between training, prior TMS recommendation experience, and TMS referral readiness.

Due to the cross‐sectional design, the direction of these associations cannot be determined. Providers who are already more comfortable with TMS may be more likely to seek training or to recommend TMS for patients, rather than training or prior TMS recommendation experience independently producing greater readiness.

High endorsement of TMS effectiveness across provider groups suggests that neuromodulation has achieved broad professional legitimacy within contemporary psychiatry, reflecting a robust evidence base and its inclusion in guidance for TRD. Despite this acceptance, many respondents reported uncertainty about referral logistics, patient counseling, and system navigation. Low referral‐pathway awareness may reflect individual knowledge gaps, limited institutional referral infrastructure, or absence of accessible TMS services in some practice settings.

### 4.1 Training Exposure and Development of Practice Readiness

Prior exposure to TMS training was associated with higher referral comfort, greater confidence in counseling patients, and better awareness of referral pathways. Attitudes were similarly high regardless of training status, suggesting that the relevance of training in this sample may relate more to operational readiness than to differences in perceived efficacy. Because of the cross‐sectional design, however, causality cannot be inferred. Training may represent a target for future intervention, but prospective studies are needed to determine whether targeted educational efforts improve actual referral behavior or readiness over time.

### 4.2 Experiential Learning and the Role of Prior TMS Recommendation Experience

The largest observed associations in this study involved prior TMS recommendation experience. Clinicians who had previously recommended TMS for patients reported greater comfort with referrals, higher counseling confidence, and better awareness of referral pathways. These associations may reflect experiential familiarity with referral workflows, but the cross‐sectional design does not establish directionality. Providers who are already more comfortable with TMS may be more likely to seek opportunities to recommend it, just as prior TMS recommendation experience may reinforce operational confidence. This pattern supports the need for future studies to test whether referral‐workflow interventions, supervised exposure, or case‐based educational strategies are associated with improvements in actual referral activity.

### 4.3 Provider Role Differences and Timing of Education

Differences by provider role point to priorities for implementation planning. Psychiatry residents endorsed TMS effectiveness at rates similar to attending psychiatrists but reported lower awareness of referral pathways. This pattern may reflect differences in educational exposure, differences in access to established referral workflows, or both. Psychiatric nurse practitioners demonstrated patterns comparable to attending psychiatrists, underscoring their role in recognizing potential candidates for neuromodulation referral within routine outpatient practice. Taken together, these findings suggest that pathway awareness may depend not only on clinician knowledge but also on whether referral processes are visible, accessible, and supported within local practice settings.

### 4.4 Perceived Barriers and System Design

Across groups, insurance coverage and cost were the most frequently endorsed barriers, followed by insufficient training exposure. This pattern suggests that system‐level ambiguity and educational gaps, rather than clinician workload, may be important obstacles to broader implementation. Practical system changes such as clarifying referral pathways and coverage criteria, standardizing documentation and orders, and adding referral guidance to the electronic health record may help reduce uncertainty. These steps could also support more consistent clinical practice, especially in larger organizations.

### 4.5 Limitations and Future Directions

Several limitations in this study warrant consideration. The overall response rate was modest (94 fully analyzable responses of 375 invitations), raising the possibility of nonresponse bias. Participants may have been more interested in neuromodulation or more favorably disposed toward TMS than nonrespondents, potentially inflating estimates of positive attitudes, counseling confidence, and referral comfort. Conversely, clinicians with limited familiarity or negative experiences may be underrepresented. Convenience sampling, with surveys distributed to attending psychiatrists and psychiatry residents and disseminated to psychiatric nurse practitioners through peer dissemination, may further limit representativeness, and the inclusion of psychiatric nurse practitioners from a single metropolitan area introduces possible coverage bias. The provider‐type difference in referral‐pathway awareness should be interpreted cautiously because it may not remain statistically significant after correction for multiple comparisons. In addition, dichotomization of Likert responses improved interpretability for the primary analyses but reduced response granularity and may have decreased statistical power. The survey instrument was not formally psychometrically validated, and the findings should be interpreted as exploratory. Finally, self‐reported outcomes are subject to social desirability and recall biases. These factors argue for caution in generalizing point estimates to all psychiatric providers in Florida.

Future research should examine whether targeted educational interventions, brief neuromodulation curricula, referral‐workflow training, and supervised exposure to TMS clinics are associated with measurable changes in referral behavior. Longitudinal designs may help clarify how preparedness evolves across residency and early faculty practice. Patient‐level outcomes also merit study, including changes in referral rates, timeliness of access, treatment initiation, and downstream clinical response. Broader, multisite studies using probability‐based sampling would improve generalizability and help delineate how institutional factors, geography, and health‐system resources shape implementation. Incorporating objective indicators such as electronic order‐set utilization, referral logs, and authorization records may help compare self‐reported readiness with observed behavior. Finally, qualitative work exploring decision‐making, uncertainties, and workflow barriers could complement survey data and help identify where implementation falters.

## 5 Conclusions

Psychiatric clinicians in this study generally viewed TMS as an effective treatment option for individuals with TRD, but operational readiness to counsel and refer was more variable. Training exposure and prior TMS recommendation experience were associated with greater confidence, comfort, and familiarity with referral pathways, although causality cannot be inferred from this cross‐sectional design.

These findings suggest that efforts to improve utilization should emphasize building operational competence and confidence, along with clearer referral workflows and accessible system‐level processes, rather than focusing only on conceptual acceptance of TMS. Future studies should test whether targeted education and referral‐workflow interventions are associated with improvements in actual referral behavior and patient access.

## Author Contributions

A.R. contributed to conceptualization, methodology, software, data curation, investigation, validation, formal analysis, supervision, visualization, project administration, resources, and writing of the original draft. L.F. contributed to investigation, validation, and writing of the original draft and participated in review and editing of the manuscript. G.W. contributed to investigation, validation, and writing of the original draft and participated in review and editing of the manuscript. C.W. contributed to conceptualization, methodology, and supervision. E.V. contributed to conceptualization, methodology, and supervision. All authors contributed to manuscript review and editing, approved the final version of the manuscript, and agree to be accountable for the work.

## Funding

The authors have nothing to report.

## Ethics Approval Statement

This study was reviewed through HCA Healthcare's centralized research compliance process and was determined to meet criteria for exemption from Institutional Review Board oversight under 45 CFR 46.104(d)(2) (reference number 2025‐1388). Exemption was granted due to the anonymous nature of the survey and use of de‐identified data. Participation was voluntary, and the study posed minimal risk. All procedures were conducted in accordance with applicable ethical standards and the Declaration of Helsinki.

## Conflicts of Interest

The authors declare no conflicts of interest.

## Supporting information




**Supplementary Material**: brb371876‐sup‐0001‐SuppMat.docx

## Data Availability

The data that support the findings of this study are available from the corresponding author upon reasonable request.

## References

[brb371876-bib-0001] Albishi, A. M. , S. A. Alhadlaq , R. T. Altowairqi , et al. 2024. “Knowledge and Attitude Toward Transcranial Magnetic Stimulation Among Rehabilitation Specialists in Saudi Arabia.” Frontiers in Bioengineering and Biotechnology 12: 1352170. 10.3389/fbioe.2024.1352170. PMC10986175 38567083

[brb371876-bib-0002] AlHadi, A. N. , A. M. AlShiban , M. A. Alomar , O. F. Aljadoa , A. M. AlSayegh , and M. A. Jameel . 2017. “Knowledge of and Attitude Toward Repetitive Transcranial Magnetic Stimulation Among Psychiatrists in Saudi Arabia.” The Journal of ECT 33, no. 1: 30–35. 10.1097/YCT.0000000000000349. PMC5321107 27564426

[brb371876-bib-0003] Avery, D. H. , P. E. Holtzheimer , W. Fawaz , et al. 2006. “A Controlled Study of Repetitive Transcranial Magnetic Stimulation in Medication‐Resistant Major Depression.” Biological Psychiatry 59, no. 2: 187–194. 10.1016/j.biopsych.2005.07.003. 16139808

[brb371876-bib-0004] Basiru, T. , H. Onyeaka , A. F. Oladunjoye , et al. 2024. “Trend and Geo‐Availability of Somatic Therapies for Treatment Resistant Depression in the US.” Psychiatry Research Communications 4: 100157. 10.1016/j.psycom.2024.100157.

[brb371876-bib-0005] Chen, L. , A. M. Fukuda , S. Jiang , et al. 2025. “Treating Depression With Repetitive Transcranial Magnetic Stimulation: A Clinician's Guide.” American Journal of Psychiatry 182, no. 6: 525–541. 10.1176/appi.ajp.20240859. PMC12323729 40302403

[brb371876-bib-0006] Cortright, M. K. , R. Bluhm , E. D. Achtyes , A. M. McCright , and L. Y. Cabrera . 2024. “Perceived Barriers to Using Neurostimulation: A National Survey of Psychiatrists, Patients, Caregivers, and the General Public.” The Journal of ECT 40, no. 2: 111–117. 10.1097/YCT.0000000000000990. PMC11136602 38265758

[brb371876-bib-0007] Deng, J. , Y. Gong , X. Lin , Y. Bao , H. Sun , and L. Lu . 2020. “Knowledge and Attitudes About Transcranial Magnetic Stimulation Among Psychiatrists in China.” BMC Psychiatry 20: 416. 10.1186/s12888-020-02817-4. PMC7444247 32831049

[brb371876-bib-0008] Fava, M. 2003. “Diagnosis and Definition of Treatment‐Resistant Depression.” Biological Psychiatry 53, no. 8: 649–659. 10.1016/S0006-3223(03)00231-2. 12706951

[brb371876-bib-0009] Gaynes, B. N. , S. W. Lloyd , L. Lux , et al. 2014. “Repetitive Transcranial Magnetic Stimulation for Treatment‐Resistant Depression: A Systematic Review and Meta‐Analysis.” The Journal of Clinical Psychiatry 75, no. 5: 477–489. 10.4088/JCP.13r08815. 24922485

[brb371876-bib-0010] Goldbloom, D. S. , and D. Gratzer . 2020. “Barriers to Brain Stimulation Therapies for Treatment‐Resistant Depression: Beyond Cost Effectiveness.” The Canadian Journal of Psychiatry 65, no. 3: 193–195. 10.1177/0706743719893584. PMC7019463 31818129

[brb371876-bib-0011] Liu, Q. , H. He , J. Yang , X. Feng , F. Zhao , and J. Lyu . 2020. “Changes in the Global Burden of Depression From 1990 to 2017: Findings From the Global Burden of Disease Study.” Journal of Psychiatric Research 126: 134–140. 10.1016/j.jpsychires.2019.08.002. 31439359

[brb371876-bib-0012] McIntyre, R. S. , M. Alsuwaidan , B. T. Baune , et al. 2023. “Treatment‐Resistant Depression: Definition, Prevalence, Detection, Management, and Investigational Interventions.” World Psychiatry 22, no. 3: 394–412. 10.1002/wps.21120. PMC10503923 37713549

[brb371876-bib-0013] Mehalick, M. L. , and G. Laje . 2020. “A Pilot Assessment of Psychiatrists' basic Knowledge and Perceptions of Transcranial Magnetic Stimulation.” Brain Stimulation 13, no. 6: 1852–1853. 10.1016/j.brs.2020.06.050.

[brb371876-bib-0014] Mrazek, D. A. , J. C. Hornberger , C. A. Altar , and I. Degtiar . 2014. “A Review of the Clinical, Economic, and Societal Burden of Treatment‐Resistant Depression: 1996–2013.” Psychiatric Services 65, no. 8: 977–987. 10.1176/appi.ps.201300059. 24789696

[brb371876-bib-0015] O'Reardon, J. P. , H. B. Solvason , P. G. Janicak , et al. 2007. “Efficacy and Safety of Transcranial Magnetic Stimulation in the Acute Treatment of Major Depression: A Multisite Randomized Controlled Trial.” Biological Psychiatry 62, no. 11: 1208–1216. 10.1016/j.biopsych.2007.01.018. 17573044

[brb371876-bib-0016] Pigott, H. E. , T. Kim , C. Xu , I. Kirsch , and J. Amsterdam . 2023. “Reanalysis of STAR*D: Cumulative Remission Rate After Four Antidepressant Trials Was 35%.” BMJ Open 13, no. 7: e074752. 10.1136/bmjopen-2022-063095 PMC10373710 37491091

[brb371876-bib-0017] Rush, A. J. , M. H. Trivedi , S. R. Wisniewski , et al. 2006. “Acute and Longer‐Term Outcomes in STAR*D: A Multistep Treatment Trial of Depression.” American Journal of Psychiatry 163, no. 11: 1905–1917. 10.1176/ajp.2006.163.11.1905. 17074942

[brb371876-bib-0018] Stern, A. P. , A. D. Boes , C. S. Haller , K. Bloomingdale , A. Pascual‐Leone , and D. Z. Press . 2016. “Psychiatrists' Attitudes toward Transcranial Magnetic Stimulation.” Biological Psychiatry 80, no. 7: e55–e56. 10.1016/j.biopsych.2015.07.027. PMC6095700 26435222

[brb371876-bib-0019] Taylor, J. J. , A. J. Manett , M. Feyder , and B. S. Bentzley . 2025. “Resource Utilization and Economic Outcomes Following Repetitive Transcranial Magnetic Stimulation for Treatment‐Resistant Depression: A Retrospective Observational Analysis.” Journal of Comparative Effectiveness Research 14, no. 7: e250019. 10.57264/cer-2025-0019. PMC12224075 40491267

[brb371876-bib-0020] Trapp, N. T. , A. Purgianto , J. J. Taylor , et al. 2025. “Consensus Review and Considerations on TMS to Treat Depression: A Comprehensive Update Endorsed by the National Network of Depression Centers, the Clinical TMS Society, and the International Federation of Clinical Neurophysiology.” Clinical Neurophysiology 170: 206–233. 10.1016/j.clinph.2024.12.015. PMC11825283 39756350

[brb371876-bib-0021] US Food and Drug Administration . 2008. “NeuroStar TMS System: De Novo Classification Request DEN070003; 510(k) K061053.” https://www.accessdata.fda.gov/scripts/cdrh/cfdocs/cfpmn/denovo.cfm?ID=DEN070003.

[brb371876-bib-0022] Vida, R. G. , E. Sághy , R. Bella , et al. 2023. “Efficacy of Repetitive Transcranial Magnetic Stimulation (rTMS) Adjunctive Therapy for Major Depressive Disorder (MDD) After Two Antidepressant Treatment Failures: Meta‐Analysis of Randomized Sham‐Controlled Trials.” BMC Psychiatry 23: 545. 10.1186/s12888-023-05033-y. PMC10375664 37501135

[brb371876-bib-0023] Yonel, H. , and M. Abdelghani . 2020. “Knowledge and Attitude towards TMS: A Brief Educational Intervention.” Progress in Neurology and Psychiatry 24, no. 1: 17–20. 10.1002/pnp.557.

[brb371876-bib-0024] Zhdanava, M. , H. Kuvadia , K. Joshi , et al. 2020. “Economic Burden of Treatment‐Resistant Depression in Privately Insured U.S. Patients With Physical Conditions.” Journal of Managed Care & Specialty Pharmacy 26, no. 8: 996–1007. 10.18553/jmcp.2020.20017. PMC10391320 32552362

