# Supplementary material for: Training and Experiential Factors Associated With Transcranial Magnetic Stimulation Referral Readiness Among Psychiatric Providers
Source: Brain Behav. 2026 Oct 30;16(11):e71876. doi: 10.1002/brb3.71876 (PMC13721900; doi:10.1002/brb3.71876)
Supplement: Supplementary file 1 [file BRB3-16-e71876-s001.docx]

**Supplemental Items: Likert Response Distributions and TMS Provider Survey**

**Supplemental Table 1. Full 5-Point Likert Response Distributions for Main Survey Items**

Distribution of 5-point Likert responses for the main survey items among the 94 analyzable respondents. Values are presented as n (%). These distributions complement the dichotomized primary analyses by preserving full response granularity. The final item assessed referral comfort using a 5-point comfort scale ranging from extremely uncomfortable to extremely comfortable.

| **Item** | **Strongly disagree** | **Somewhat disagree** | **Neither** | **Somewhat agree** | **Strongly agree** |
| --- | --- | --- | --- | --- | --- |
| I understand the clinical indications and contraindications for TMS | 11 (11.7%) | 23 (24.5%) | 8 (8.5%) | 22 (23.4%) | 30 (31.9%) |
| I know the appropriate referral process for patients to receive TMS in my setting | 11 (11.7%) | 10 (10.6%) | 23 (24.5%) | 22 (23.4%) | 28 (29.8%) |
| I believe TMS is an effective treatment option for certain people suffering from depression | 1 (1.1%) | 2 (2.1%) | 5 (5.3%) | 36 (38.3%) | 50 (53.2%) |
| I feel confident discussing TMS as a treatment option with patients | 2 (2.1%) | 7 (7.4%) | 10 (10.6%) | 38 (40.4%) | 37 (39.4%) |
| Insurance coverage limitations prevent me from recommending TMS to some patients | 5 (5.3%) | 13 (13.8%) | 14 (14.9%) | 31 (33.0%) | 31 (33.0%) |
| Cost considerations are a significant barrier to TMS utilization in my clinical practice | 3 (3.2%) | 8 (8.5%) | 18 (19.1%) | 33 (35.1%) | 32 (34.0%) |
| Lack of patient interest or misconceptions about TMS is a significant barrier | 6 (6.4%) | 11 (11.7%) | 33 (35.1%) | 30 (31.9%) | 14 (14.9%) |
| I need further training or education regarding TMS to feel fully confident recommending TMS | 6 (6.4%) | 11 (11.7%) | 11 (11.7%) | 26 (27.7%) | 40 (42.6%) |
| I am comfortable referring patients for TMS | 2 (2.1%) | 10 (10.6%) | 6 (6.4%) | 33 (35.1%) | 43 (45.7%) |

**TMS Provider Survey**

**Transcranial Magnetic Stimulation (TMS) Provider Perceptions Survey**

Instructions:
For each statement, please indicate your level of agreement by circling a number:
1 = Strongly Disagree

2 = Disagree

3 = Neither Agree nor Disagree

4 = Agree

5 = Strongly Agree

**Section 1: Survey Consent**

1. Provider survey Information and Consent
 □ Yes
 □ No

**Section 2: Demographics**

2. Occupation (select one):

□ Psychiatrist
 □ Resident psychiatrist

□ Psychiatric nurse practitioner

3.Years in practice:
 □ 0–5
 □ 6–10
 □ 11–15
 □ 16–20
 □ >20

4. Primary practice setting:
 □ Academic medical center
 □ Private practice
 □ Community health center
 □ Hospital system
 □ Other: __________

1. Practice location:
    □ Urban
    □ Suburban
    □ Rural
2. Estimated number of depression patients seen monthly:
    □ <10
    □ 10–25
    □ 26–50
    □ 51–100
    □ >100

**Section 3: Knowledge and Experience**

3. Have you previously recommended TMS for a patient?

Circle one: Yes / No

4. I have received formal education or training on TMS and its clinical indications.

Circle one: 1	2	3	4	5

5. I understand the clinical indications and contraindications for TMS.

Circle one: 1	2	3	4	5

6. I know the appropriate referral process for patients to receive TMS in my setting.

Circle one: 1	2	3	4	5

**Section 4: Attitudes Toward TMS Efficacy and Safety**

7. I believe TMS is an effective treatment option for certain people suffering from depression.

Circle one: 1	2	3	4	5

8. I feel confident discussing TMS as a treatment option with patients.

Circle one: 1	2	3	4	5

9. I believe TMS is comparably effective to other depression treatments (e.g., medication, therapy) in appropriate cases.

Circle one: 1	2	3	4	5

**Instructions:
For statement #14, please indicate your level of comfort by circling a number:**
1 = Extremely Uncomfortable

2 = Somewhat Uncomfortable

3 = Neither Comfortable or Uncomfortable

4 = Somewhat Comfortable

5 = Extremely Comfortable

10. I am comfortable referring appropriate patients for TMS.

Circle one: 1	2	3	4	5

11. I am familiar with the safety profile and side effects of TMS.

Circle one: 1	2	3	4	5

**Section 5: Implementation, Setting, and Access**

12. TMS is accessible for patients in my practice setting or community.

Circle one: 1	2	3	4	5

13. My practice or institution provides adequate education/resources about TMS.

Circle one: 1	2	3	4	5

14. TMS referrals are encouraged or supported in my practice setting.

Circle one: 1	2	3	4	5

15. There are sufficient local resources to refer patients for TMS.

Circle one: 1	2	3	4	5

16. Distance to the nearest TMS provider is NOT a major barrier for my patients.

Circle one: 1	2	3	4	5

**Section 6: Barriers to TMS**

17. Insurance coverage limitations prevent me from recommending TMS to some patients.

Circle one: 1	2	3	4	5

18. Cost considerations are a significant barrier to TMS utilization in my clinical practice.

Circle one: 1	2	3	4	5

19. Lack of patient interest or misconceptions about TMS is a significant barrier.

Circle one: 1	2	3	4	5

20. I need further training or education regarding TMS to feel fully confident recommending it.

Circle one: 1	2	3	4	5
